# Evolutionary analysis of exogenous and integrated HHV-6A/HHV-6B populations

**DOI:** 10.1093/ve/veaa035

**Published:** 2020-04-30

**Authors:** Diego Forni, Rachele Cagliani, Mario Clerici, Uberto Pozzoli, Manuela Sironi

**Affiliations:** v1 Bioinformatics, Scientific Institute IRCCS E. MEDEA, 23842 Bosisio Parini, Lecco, Italy; v2 Department of Physiopathology and Transplantation, University of Milan, 20090 Milan, Italy; v3 IRCCS Fondazione Don Carlo Gnocchi, 20148 Milan, Italy

**Keywords:** HHV-6, Human betaherpesvirus, population structure, viral evolution, Roseolovirus

## Abstract

Human betaherpesviruses 6A and 6B (HHV-6A and HHV-6B) are highly prevalent in human populations. The genomes of these viruses can be stably integrated at the telomeres of human chromosomes and be vertically transmitted (inherited chromosomally integrated HHV-6A/HHV-6B, iciHHV-6A/iciHHV-6B). We reconstructed the population structures of HHV-6A and HHV-6B, showing that HHV-6A diverged less than HHV-6B genomes from the projected common ancestral population. Thus, HHV-6B genomes experienced stronger drift, as also supported by calculation of nucleotide diversity and Tajima’s *D*. Analysis of ancestry proportions indicated that HHV-6A exogenous viruses and iciHHV-6A derived most of their genomes from distinct ancestral sources. Conversely, ancestry proportions were similar in exogenous HHV-6B viruses and iciHHV-6B. In line with previous indications, this suggests the distinct exogenous viral populations that originated iciHHV-6B in subjects with European and Asian ancestry are still causing infections in the corresponding geographic areas. Notably, for both iciHHV-6A and iciHHV-6B, we found that European and American sequences tend to have high proportions of ancestry from viral populations that experienced considerable drift, suggesting that they underwent one or more bottlenecks followed by population expansion. Finally, analysis of HHV-6B exogenous viruses sampled in Japan indicated that proportions of ancestry components of most of these viruses are different from the majority of those sampled in the USA. More generally, we show that, in both viral species, both integrated and exogenous viral genomes have different ancestry components, partially depending on geographic location. It would be extremely important to determine whether such differences account for the diversity of HHV-6A/HHV-6B-associated clinical symptoms and epidemiology. Also, the sequencing of additional exogenous and integrated viral genomes will be instrumental to confirm and expand our conclusions, which are based on a relatively small number of genomes, sequenced with variable quality, and with unequal sampling in terms of geographic origin.

## 1. Introduction

Human betaherpesviruses 6A and 6B (HHV-6A and HHV-6B) are members of the *Roseolovirus* genus, in the *Betaherpesviridae* subfamily. Initially described as viral variants, HHV-6A and HHV-6B are now formally recognized as distinct species by the International Committee on Taxonomy of Viruses and reportedly display about 90 per cent interspecies genome sequence identity (Ablashi et al. 2014). HHV-6A and HHV-6B genomes comprise a long unique region (∼140 to 143 kb) flanked by identical direct repeats (DR, ∼8 to 8.5 kb each) (Gompels et al. 1995; Dominguez et al. 1999; Krug and Pellett 2014; Tweedy et al. 2015a). A variable number of perfect and imperfect copies of the hexameric human telomeric repeats are located at the right and left ends of the DRs and are thought to mediate viral integration into the host genome (Gompels et al. 1995; Dominguez et al. 1999; Krug and Pellett 2014). In fact, unlike all other human herpesviruses, HHV-6A and HHV-6B, as well as HHV-7, can integrate and persist in the telomeric regions of host chromosomes (Luppi et al. 1993; Nacheva et al. 2008; Prusty et al. 2017).

Where studied, primary infection with HHV-6B is extremely common and generally occurs before twenty-four months of age. The most common symptom is high fever, although a minority of patients develop other symptoms including rash, seizures, and encephalitis (Hill and Zerr 2014; Tesini, Epstein, and Caserta 2014). For unknown reasons, the frequency of neurological complications varies with geography, being particularly high in Japan: whereas encephalitis due to primary HHV-6B infection is rarely reported in the USA and Europe, estimates suggest that its prevalence in Japan is 5.5/100,000 infected children (Yoshikawa et al. 2009; Tesini, Epstein, and Caserta 2014; Ward 2014). The epidemiology of HHV-6A is less well understood. Compared to HHV-6B, primary infection has been reported to occur later in life in Europe, the USA, and Asia. HHV-6A infant infections have been reported in Sub-Saharan Africa at both common and low prevalence in different patient or healthy populations (Kasolo, Mpabalwani, and Gompels 1997; Bates et al. 2009; Tembo et al. 2015).

After primary infection, HHV-6A/HHV-6B achieves latency, possibly by chromosomal integration in a small number of host cells (Arbuckle et al. 2010). Reactivation of latent HHV-6B represents a leading cause of central nervous system disease in transplant recipients (Hill and Zerr 2014).

HHV-6A/HHV-6B chromosomal integration can also occur in the germline: in this case the viral genome is vertically transmitted from parent to child, behaving as a Mendelian trait (inherited chromosomally integrated HHV-6A/HHV-6B, iciHHV-6A/iciHHV-6B) (Daibata et al. 1999; Tanaka-Taya et al. 2004; Kaufer and Flamand 2014). The proportion of iciHHV-6A/iciHHV-6B carriers in the general population is 0.2–1 per cent, with possible geographic differences in both overall frequency and the relative proportion of HHV-6A and HHV-6B integrations (Clark 2016; Tweedy et al. 2016).

Besides complicating the diagnosis of active HHV-6A/HHV-6B infection, reactivation of integrated virus was documented during pregnancy (Gravel, Hall, and Flamand 2013) and in an immunocompromised child (Endo et al. 2014). iciHHV-6A/iciHHV-6B carriage status has been associated with cardiac disease (Das 2015; Gravel et al. 2015; Kuhl et al. 2015; Tweedy et al. 2015b) and with higher risk of acute graft versus host disease in hematopoietic stem cell transplant recipients (Hill et al. 2017). Whether iciHHV-6A/iciHHV-6B has additional consequences for human health is still unclear (Clark 2016; Collin and Flamand 2017). Likewise, it is presently unknown whether viral genetic determinants modulate disease presentation and geographic differences in clinical symptoms and epidemiology. Indeed, it was not until very recently that complete sequences for more than 180 HHV-6A/HHV-6B genomes were obtained (Tweedy et al. 2016; Zhang et al. 2017; Greninger et al. 2018a,b; Telford, Navarro, and Santpere 2018). Analyses indicated that HHV-6A genomes are more diverse than HHV-6B (Tweedy et al. 2015b, 2016; Telford, Navarro, and Santpere 2018) and that iciHHV-6A are divergent from exogenous HHV-6A viruses (Tweedy et al. 2016; Zhang et al. 2017). Generally, iciHHV-6B are highly similar to circulating HHV-6B viruses and several iciHHV-6B genomes were found to be nearly identical, suggesting that they derived from the same integration event (Tweedy et al. 2015b, 2016; Zhang et al. 2017; Greninger et al. 2018a). By mapping integration sites, Zhang et al. (2017) indicated that this is the case for five iciHHV-6B sequenced from European subjects and estimated that their common ancestor existed long ago (∼24,000 years ago). Based on sequence divergence, an earlier origin for iciHHV-6A than for iciHHV-6B was also proposed (Tweedy et al. 2015b, 2016).

Some level of geographic clustering was also reported for HHV-6A and HHV-6B (Tweedy et al. 2016; Zhang et al. 2017; Greninger et al. 2018a; Telford, Navarro, and Santpere 2018; Peddu et al. 2019), but the evolutionary history of these viruses has remained largely unexplored.

## 2. Materials and methods

### 2.1 Sequences, alignments, and recombination

We retrieved sequences of complete or almost complete HHV-6A and HHV-6B genomes from the NCBI database (http://www.ncbi.nlm.nih.gov/). Only one viral genome was retained from each set of inherited chromosomally integrated sequences derived from first-degree relatives (Greninger et al. 2018a), leading to a final set of 181 sequences (a list of NCBI accession number is reported in Supplementary Table S1).

HHV-6A strains included ten exogenous viruses and nineteen inherited chromosomally integrated sequences, whereas HHV-6B genomes were composed of sixty-eight viruses and eighty-four integrated genomes. Viral genomes were classified as integrated or exogenous based on how they were classified in the original works (see Supplementary Table S1) (Gompels et al. 1995; Isegawa et al. 1999; Tweedy et al. 2015a; Zhang et al. 2017; Greninger et al. 2018a,b; Telford, Navarro, and Santpere 2018). Whole genome sequence alignments were generated using MAFFT (Katoh and Standley 2013) with the default parameters.

From complete genome alignments of all HHV-6A/HHV-6B, HHV-6A, and HHV-6B sequences, 14,643, 4,687, and 2,810 parsimony-informative (PI) sites (sites that contain at least two types of nucleotides, each with a minimum frequency of two) were obtained, respectively. PIs were only included if sequence information (non-gap nucleotide) was available for at least 50 per cent of sequences. These sites were used as input for discriminant analysis of principal components (DAPC), linkage disequilibrium (LD), and population structure analyses.

Recombination was evaluated using four methods implemented in RDP4 (RDP, GENECONV, MaxChi, and Chimera) (Sawyer 1989; Smith 1992; Martin and Rybicki 2000; Posada and Crandall 2001; Martin et al. 2017). These methods were used because they showed good power in previous simulation analyses (Posada and Crandall 2001; Bay and Bielawski 2011).

In particular, the analysis was performed on the HHV-6A/HHV-6B sequence alignment, after removing small repeats annotated as ‘rpt_type=TANDEM’ in either the HHV-6A or HHV-6B reference genomes (NC_001664 and NC_000898). To be conservative, only recombination events longer than 500 bp, with known parental sequences, and with a P value for all four methods <0.01 were considered as significant. RDP4 was run with general default parameters and no permutations, by setting sequences as linear, by requiring topological evidence, and by checking alignment consistency. For the four methods we applied, default parameters were also used (RDP: widow size = 30; MaxChi: number of variable sites per window = 70, ‘strip gap’ option on; Chimera: number of variable sites per window = 60; GENECONV: ‘treat indel blocks as one polymorphism’ option on).

### 2.2 Network construction and DAPC analysis

A neighbor-net split network of all whole genome sequences was constructed with SplitsTree v4.13.1 (Huson and Bryant 2006) using uncorrected *p*-distances and all polymorphic sites, after removing gap sites.

A DAPC (Jombart, Devillard, and Balloux 2010) was applied for all PIs in the HHV-6A/HHV-6B alignment. DAPC was selected because it allows the identification of clusters of genetically related individuals. Whereas approaches such as PCA (principal component analysis) describe the global diversity in a sample or population, in DAPC variance is partitioned into a between-cluster and within-cluster component. DAPC thus searches for synthetic variables (allele combinations) that maximize the variation among clusters while minimizing the variation within clusters. In DAPC, data are first transformed using PCA (to reduce the number of variables), the optimal number of clusters is identified, and then each sample is assigned to one of these clusters. The best number of clusters (*K* = 13) was identified through a Bayesian Information Criterion (Supplementary Fig. S1), using a sequential *K*-means clustering method with *K* from 1 to 15. After that, a discriminant analysis of the number of principal components that explained more than 95 per cent of variance was applied, and clusters of the first and second linear discriminants were plotted. DAPC was carried out with the Adegenet R package (Jombart 2008).

### 2.3 Linkage disequilibrium and population structure

LD—that is the non-random association of alleles at different polymorphic positions—was evaluated using the LIAN software v3.7 (Haubold and Hudson 2000). Briefly, LIAN tests for independent assortment by computing the number of loci at which each pair of haplotypes differs. From the distribution of mismatch values the program calculates a variance (*V*_D_), which is then compared to the variance expected under linkage equilibrium (*V*_e_). The null hypothesis that *V*_D_ = *V*_e_ can be tested by Monte Carlo simulation: the original dataset is scrambled by resampling the loci without replacement and for each resampled dataset, the *V*_D_ value is computed. The significance of the difference between *V*_D_ and *V*_e_ is calculated as the proportion of resampled datasets with a *V*_D_ value greater than or equal to that of the original dataset. We used this procedure with 1,000 iterations. LIAN also computes a standardized index of association (*I*_A_^S^), which is a measure of linkage and is based on the ratio between *V*_D_ and *V*_e_ scaled by the number of polymorphic positions. Such scaling has the advantage of making *I*_A_^S^ comparable between studies (Hudson 1994; Haubold and Hudson 2000). The *I*_A_^S^ is zero for linkage equilibrium.

We analyzed the population structure of HHV-6A/HHV-6B using the program STRUCTURE (version 2.3) (Pritchard, Stephens, and Donnelly 2000). In general, population structure refers to a deviation from panmixia, whereby populations are somehow subdivided into subpopulations (e.g. due to geographic isolation). Very often subpopulations display differences in allele frequencies and the STRUCTURE method relies on a model in which the whole population is subdivided into *K* subpopulations characterized by a set of allele frequencies at each locus (Pritchard, Stephens, and Donnelly 2000). To run STRUCTURE, the allele frequency spectrum parameter (*λ*) was first estimated by using the ‘estimate *λ*’ model for *K* = 1, as suggested (Falush, Stephens, and Pritchard 2003): values of 0.58 (all HHV-6A/HHV-6B), 1.49 (HHV-6A), and 0.40 (HHV-6B) were obtained and used in the subsequent analyses. We next applied the linkage model with correlated allele frequencies, which extends the admixture model to (weakly) linked loci (Falush, Stephens, and Pritchard 2003). The admixture model with correlated frequencies allows for individuals to have mixed ancestry and assumes allele frequencies in closely related subpopulations to be similar because of migration or shared ancestry. Thus, this model has good power to detect subtle population structures (Falush, Stephens, and Pritchard 2003). The admixture model with correlated frequencies also assumes that all subpopulations diverged from a common ancestral population, which is characterized by a set of allele frequencies estimated by the model. The amount of drift that each subpopulation experienced from these ancestral frequencies is quantified by the *F* parameter, which is thus similar in concept to the fixation index (*F*_ST_), a measure of population differentiation based on allele frequencies.

To run STRUCTURE, map distances were set equal to PI site physical distances. The optimal number of populations was determined by running the model for *K*=1 to *K*=10. For each *K*, ten runs were performed with MCMC run lengths of 50,000 and 20,000 burn-in. Evanno's method (also known as the Δ*K* method) was used to select the optimal *K* (Evanno, Regnaut, and Goudet 2005). Results of independent runs were merged by permutating clusters using CLUMPAK (Kopelman et al. 2015) to generate the *Q*-value matrix.

To assess the effect of sample size on the *F* parameter, ten runs of STRUCTURE were performed for *K* = 2. Specifically, for each run, ten HHV-6B exogenous viral genomes (as the number of exogenous HHV-6A genomes) and nineteen iciHHV-6B (as the number of iciHHV-6A) were randomly selected.

### 2.4 Nucleotide diversity

Nucleotide diversity on whole genome alignments was estimated using two parameters: the scaled number of segregating sites (*θ*_W_ (Watterson 1975)) and the average number of pairwise differences (*π* (Nei and Li 1979)). Tajima’s *D* (Tajima 1989) was also calculated. All analyses were performed using the POP-GENOME R package (Pfeifer et al. 2014).

## 3. Results

### 3.1 Phylogenetic relationships and DAPC

**Figure 1.**
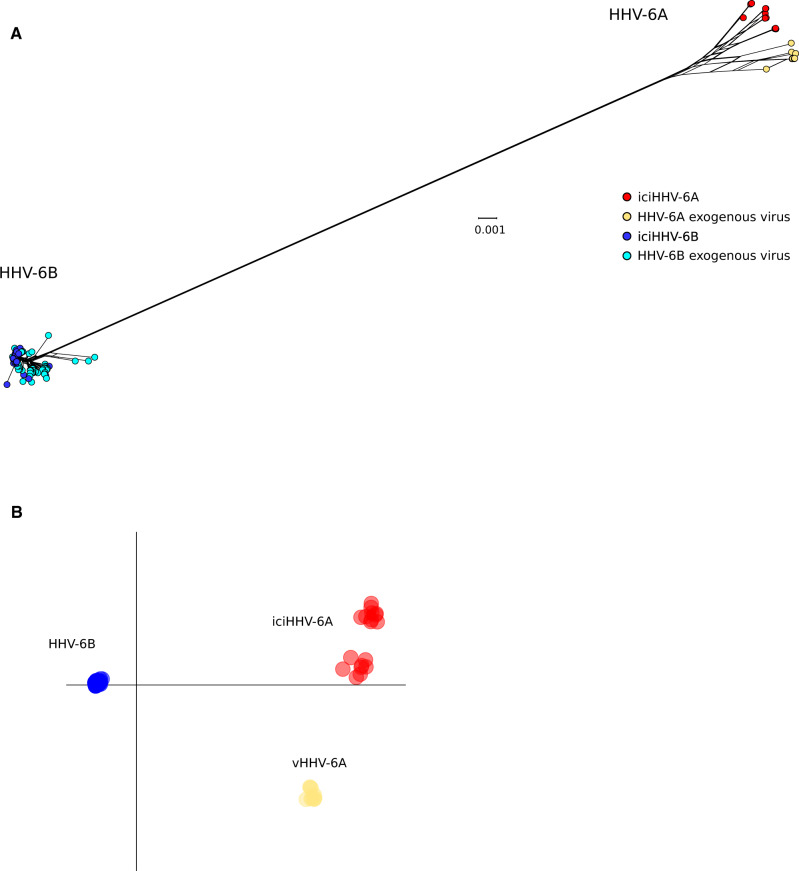
HHV-6A/HHV-6B divergence. (A) Neighbor-net split network of 181 HHV-6A/HHV-6B genome sequences. Each sample is shown as a dot and colors are indicated in the legend. (B) DAPC of HHV-6A/HHV-6B genomes. The first two discriminants are plotted and samples are colored as in panel A. iciHHV-6A/iciHHV-6B, inherited chromosomally integrated HHV-6A and HHV-6B; vHHV-6A, HHV-6A exogenous virus.

We obtained a list of complete or almost complete HHV-6A/HHV-6B genomes from public databases. These 181 genomes (twenty-nine classified as HHV-6A and 152 as HHV-6B) were aligned and a neighbor-net split network was generated. As expected, HHV-6A and HHV-6B sequences were clearly separated in the network (Fig. 1A). HHV-6A sequences showed higher genetic diversity, especially between iciHHV-6A and exogenous viruses, whereas most HHV-6B genomes were very closely related and clustered together.

To gain further insight into the structure of HHV-6A/HHV-6B populations, we applied a DAPC. This approach can identify the principal components that explain most between‐group variation while minimizing within‐group variation (Jombart, Devillard, and Balloux 2010).

Results showed the presence of four major clusters, reflecting the neighbor-net split network (Fig. 1B). HHV-6A and HHV-6B were clearly separated, with the former generating three distinct clusters, two composed of iciHHV-6A sequences, and the other including exogenous viruses only. The integration sites are known for only three iciHHV-6A and they involve three different chromosomes (19, 10, and 17) (Nacheva et al. 2008; Zhang et al. 2016). Thus, the two iciHHV-6A clusters we observed cannot merely be accounted for by the inclusion of sequences deriving from the same common ancestral events. Conversely, most of HHV-6B genomes, both integrated copies and exogenous viruses, clustered together (Fig. 1B).

### 3.2 Linkage disequilibrium and recombination

Intraspecies recombination was previously documented for HHV-6A and HHV-6B (Tweedy et al. 2015b; Greninger et al. 2018a; Telford, Navarro, and Santpere 2018), whereas only one interspecies recombinant (the DA strain) has been reported to date and proposed to have originated via co-culturing of HHV-6A and HHV-6B viruses (Greninger et al. 2018b). We thus first analyzed the level of LD with LIAN v3.7, which tests the null hypothesis of linkage equilibrium across loci (Haubold and Hudson 2000). Statistically significant LD was detected for both HHV-6A and HHV-6B sequences (Monte Carlo simulations, 1,000 repetitions, P < 10^−3^). However, the *I*_A_^S^ resulted equal to 0.199 for HHV-6A and 0.08 for HHV-6B. *I*_A_^S^ is a multilocus measure of LD: it is equal to zero in the case of free recombination (linkage equilibrium). The values for HHV-6A and HHV-6B indicate low to moderate LD and are, therefore, consistent with some level of recombination. As a comparison, the *I*_A_^S^ for other recombining viruses such as HIV-1 and HBV (Arenas et al. 2018) is around 0.04, whereas it amounts to values between 0.14 and 0.5 for viruses that recombine infrequently or in which recombination only occurs at the intra-genotype (i.e. HCV and Dengue virus) (Holmes and Twiddy 2003; Szmaragd and Balloux 2007). As expected, LD was higher when HHV-6A and HHV-6B were analyzed together (*I*_A_^S^ = 0.507).

We next directly explored whether interspecies recombination occurred using four methods implemented in the RDP4 software. To be conservative, we only retained recombination events longer than 500 bp and with known parental sequences. Several intraspecies events were detected, for both HHV-6A and HHV-6B (not shown). However, the only interspecies recombination event was the one previously described for the DA strain (Greninger et al. 2018b).

### 3.3 Population structure analysis

To gain further insight into the evolutionary origin of HHV-6A and HHV-6B genomes, we investigated ancestry and admixture using the STRUCTURE program, which relies on a Bayesian statistical model for clustering genotypes into populations without prior information on their genetic relatedness or geographic origin. STRUCTURE can identify distinct subpopulations (or clusters, *K*) that compose the overall population and assigns each individual to one or more of those clusters. Initially, the 181 HHV-6A and HHV-6B genomes were used for STRUCTURE analysis with correlated allele frequencies under the linkage model (Pritchard, Stephens, and Donnelly 2000; Falush, Stephens, and Pritchard 2003). This model, which is well suited for markers that are weakly linked (Falush, Stephens, and Pritchard 2003), uses LD among loci and assumes that discrete genome ‘chunks’ are inherited from ancestral populations.

**Figure 2.**
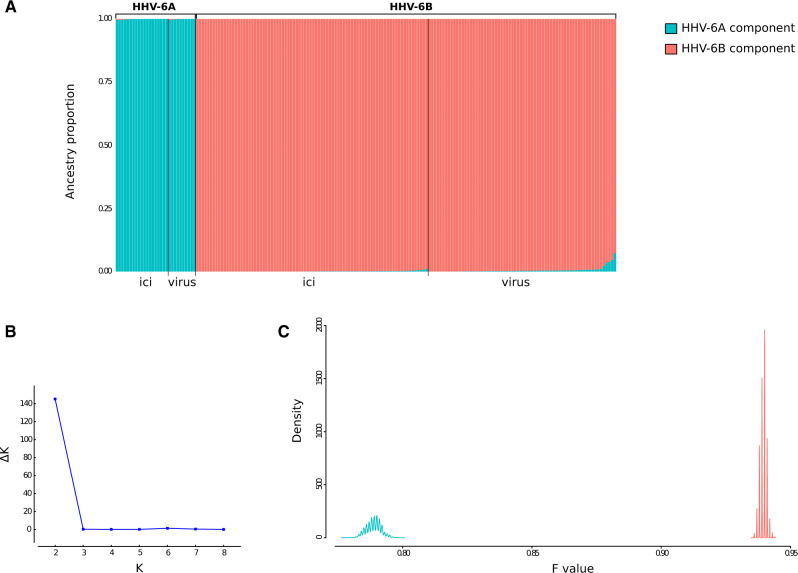
HHV-6A/HHV-6B population structure. (A) Bar plot representing the proportion of ancestral population components from the STRUCTURE linkage model for all HHV-6A/HHV-6B genomes. Each vertical line represents a HHV-6A/HHV-6B genome. Samples are ordered on the basis of their integration/exogenous status and of viral species classification. (B) Analysis of optimal *K* for STRUCTURE analysis. Δ*K* is calculated as Δ*K* = mean(|*L*''(*K*)|)/SD(*L*(*K*)). The peak of this distribution is the optimal *K* used in STRUCTURE analysis. (C) Distributions of posterior *F* values for the ancestral populations. Colors are as in panel A.

The optimal number of subpopulations (*K*) was estimated to be equal to 2 and STRUCTURE clearly separated the HHV-6A and HHV-6B ancestry components (Fig. 2A and B). A minority of HHV-6B sequences displayed low levels of HHV-6A ancestry component; this latter is accounted for by different chunks distributed across the genomes (Supplementary Fig. S2). The linkage model also provides information on the level of drift of each subpopulation from a hypothetical common ancestral population. In particular, STRUCTURE estimates the *F* parameter, which represents a measure of genetic differentiation between populations based on allele frequencies (see Section 2). The *F* value for the HHV-6A component was substantially lower than that of the HHV-6B component (Fig. 2C), indicating that HHV-6A genomes diverged less than HHV-6B genomes from the ancestral common HHV-6A/HHV-6B population. This conclusion is not influenced by the different sample size of HHV-6A and HHV-6B genomes, as ten random resamplings of a number of HHV-6B sequences equal to that of HHV-6A provided the same result (Supplementary Fig. S3).

The strong genetic differentiation between the HHV-6A and HHV-6B populations may mask further sub-level clustering. Thus, STRUCTURE analysis was repeated for HHV-6A and HHV-6B genomes separately.

**Figure 3.**
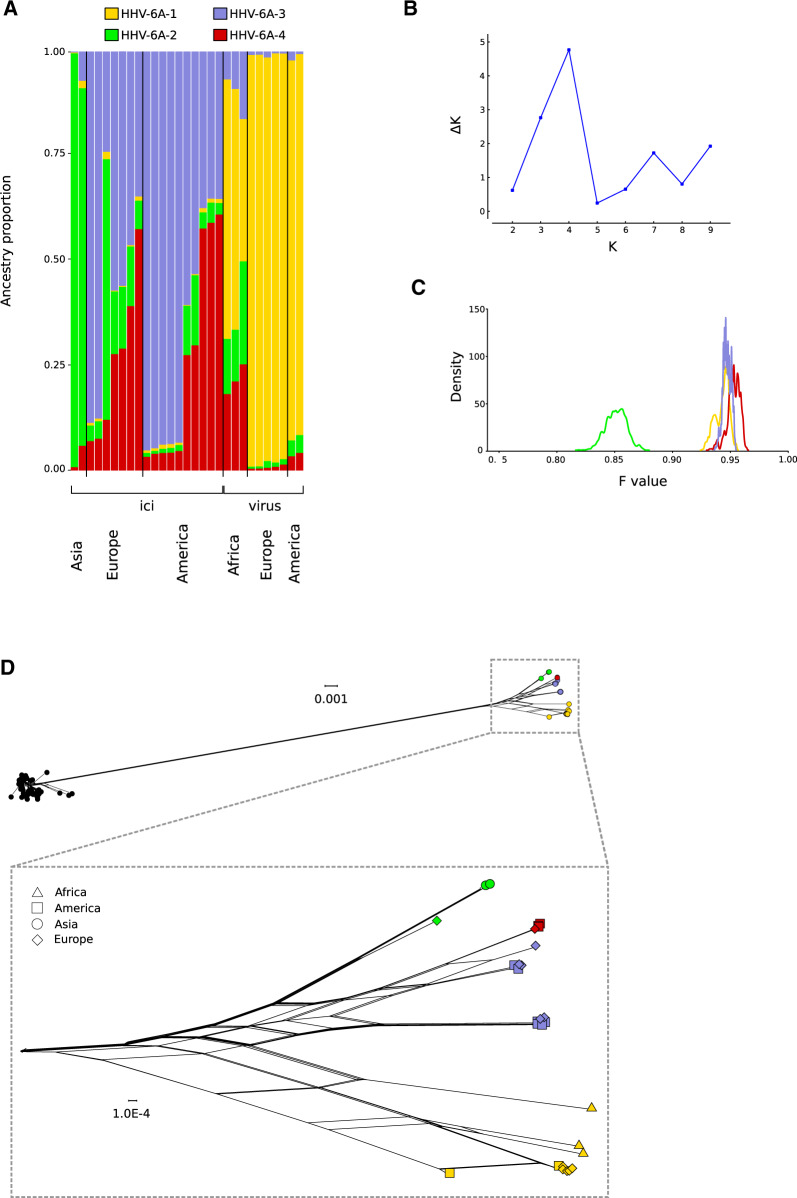
HHV-6A population structure. (A) Bar plot representing the proportion of ancestral population components from the STRUCTURE linkage model for HHV-6A genomes. Each vertical line represents a HHV-6A genome. Samples are ordered on the basis of their integration/exogenous status and of geographic origin. (B) Analysis of optimal *K* for STRUCTURE. *K*= 4 was used in the analysis. (C) Distributions of posterior *F* values for the ancestral populations. Colors are as in panel A. (D) Neighbor-net split network of 181 HHV-6A/HHV-6B genome sequences. Tips (sequences) are colored according to the major ancestry component identified by STRUCTURE. In the enlargement of HHV-6A, tip shapes denote geographic origin.

For HHV-6A, the best *K* resulted equal to 4 (Fig. 3A and B). Analysis of ancestry components indicated that a large proportion (almost 100% for non-African viruses) of exogenous viral genomes was contributed by a single ancestral population (population HHV-6A-1, Fig. 3A). The European exogenous viruses were all originally isolated in 1991 in Germany from patients with collagen vascular disease (Krueger et al. 1991), whereas the two American samples (DA and GS strains) were isolated in the same laboratory in the 80s (Salahuddin et al. 1986; Ablashi et al. 1988). The HHV-6A-1 component was virtually absent in iciHHV-6A and these latter showed limited evidence of geographic structure and variable levels of admixture among three ancestral populations, with the exclusion of the two Asian sequences (one from China and one from Japan), which had a major proportion of their ancestry from a single population (population HHV-6A-2, Fig. 3A). In turn, population HHV-6A-2 contributed a proportion of ancestry to iciHHV-6A sampled in Europe and the USA, as well as to African HHV-6A viruses. The major ancestry components inferred by STRUCTURE clearly discriminated sequence clusters in the network (Fig. 3D). The *F* value for population HHV-6A-2 was substantially lower than those for the three other populations (Fig. 3C). The homogeneous ancestry of European and American exogenous viruses suggests the population underwent a bottleneck. Overall, these results are consistent with the DAPC and splits-tree analysis by showing that HHV-6A exogenous viruses and iciHHV-6A are divergent and derived most of their genomes from distinct ancestral populations.

**Figure 4.**
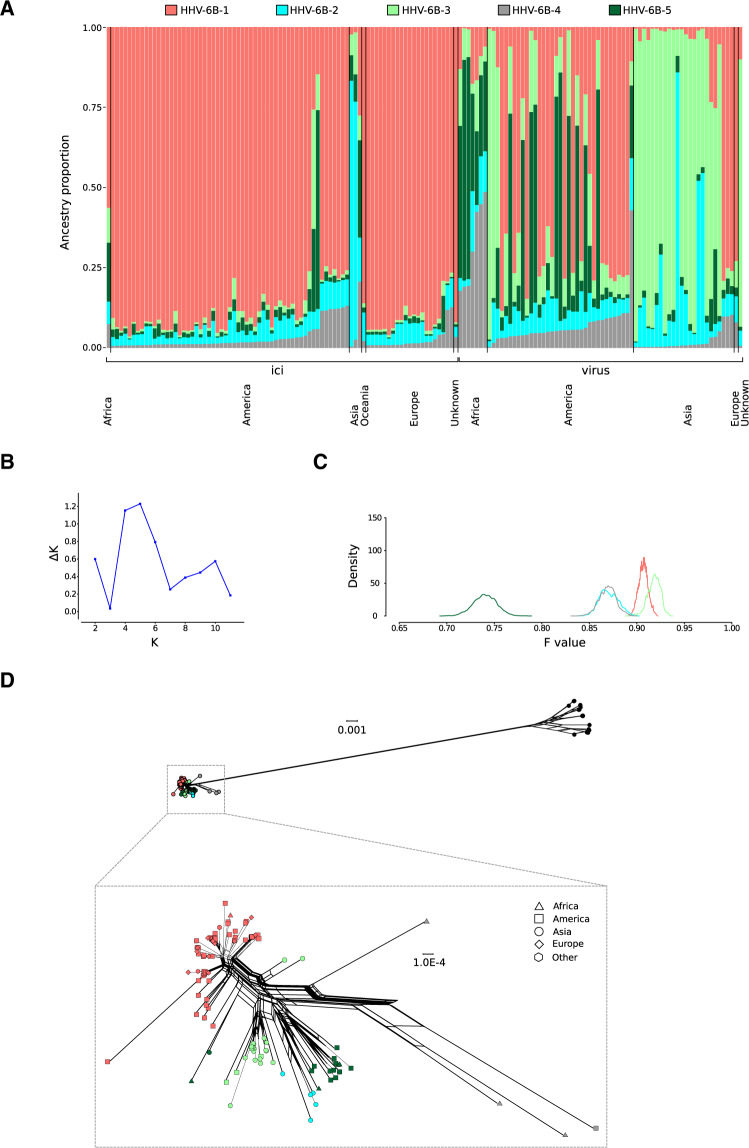
HHV-6B population structure. (A) Bar plot representing the proportion of ancestral population components from the STRUCTURE linkage model for HHV-6B genomes. Each vertical line represents a HHV-6B genome. Samples are ordered on the basis of their integration/exogenous status and of geographic origin. (B) Analysis of optimal *K* for STRUCTURE. *K* = 5 was used in the analysis. (C) Distributions of posterior *F* values for the ancestral populations. Colors are as in panel A. (D) Neighbor-net split network of 181 HHV-6A/HHV-6B genome sequences. Tips (sequences) are colored according to the major ancestry component identified by STRUCTURE. In the enlargement of HHV-6B, tip shapes denote geographic origin.

In the case of HHV-6B, the best number of cluster was estimated to be 5, although a similar Δ*K* was obtained for *K* = 4 (Fig. 4A and B). Plotting of five ancestral components (populations HHV-6B-1 to HHV-6B-5) indicated that they were represented in both exogenous viruses and iciHHV-6B, although with very different proportions, also depending on geographic location (Fig. 4A). Very similar results were obtained when data for *K* = 4 were analyzed (Supplementary Fig. S4). Most iciHHV-6B sampled in Europe (from the UK, Italy, and Finland) and the USA showed similar and low levels of admixture. The major ancestry component (HHV-6B-1) of these genomes had one of the highest *F* values (Fig. 4C). This suggests that the exogenous viral populations that integrated into these chromosomes had already drifted away from the ancestral common population, most likely as the result of a bottleneck. Such an exogenous viral population is still being actively transmitted in the USA and Europe, as several exogenous viruses have very similar ancestry components as American (all of them from New York state) and European (a single genome from France) iciHHV-6B (Fig. 4A) (Aubin et al. 1991; Greninger et al. 2018a). Conversely, two Asian iciHHV-6B acquired a major proportion of their genomes from another population (HHV-6B-2). This population, which experienced relatively limited drift, also accounts for a large ancestry proportion of some Asian exogenous viruses (Fig. 4A), again suggesting that the viral population that originated the iciHHV-6B integration events is still circulating in Asia. Interestingly, though, most Asian exogenous viruses (all of them from Japan) had a major ancestry component from population HHV-6B-3, with the highest *F* value and poorly represented elsewhere (with the exclusion of two American viruses) (Fig. 4A and C). Again, this suggests that the Asian viruses with high HHV-6B-3 ancestry proportions experienced a bottleneck. Finally, exogenous viruses sampled in Africa had admixed genomes with high proportions derived from the three ancestral populations that experienced limited drift (Fig. 4A and C). The major ancestry components inferred by STRUCTURE agreed with the clustering obtained with the neighbor-net split network (Fig. 4D).

### 3.4 Levels and patterns of intraspecies polymorphism

Finally, to further investigate the evolutionary history of HHV-6A and HHV-6B, the level of intraspecies polymorphism was examined by estimating genetic diversity (*θ*_w_ and *π*) at all polymorphic sites (Watterson 1975; Nei and Li 1979). Tajima’s *D* (Tajima 1989) was also calculated. *D* values close to zero are expected in neutrally evolving populations of constant size, whereas positive values derive from an excess of intermediate frequency variants, due to balancing selection or population shrinkage. Negative Tajima’s *D* values indicate an excess of low frequency polymorphism caused either by background selection, selective sweeps or population expansion. Although the effect of demography and selection are difficult to disentangle, selective events usually affect a relatively small fraction of sites and genome-wide estimates are thus more likely to be indicative of demographic forces acting on the population.

The highest nucleotide diversity was observed for exogenous and integrated HHV-6A (Table 1). However, iciHHV-6A had positive values of Tajima's *D* relatively close to 0, suggesting almost constant population size. HHV-6A exogenous viruses also had relatively high levels of polymorphism; taking the STRUCTURE results into account, this is likely contributed by the African viruses. Nonetheless, Tajima's *D* was negative, suggesting that the European and American viruses underwent a bottleneck and recent population expansion.

**Table 1.** Nucleotide diversity and Tajima’s *D*.

Sequence set	Number of sequences	*θ_w_* (×10^−3^)	*π* (×10^−3^)	Tajima’s *D*
ici-HHV-6A	19	2.83	2.98	0.38
vHHV-6A	10	2.66	1.93	−1.38
iciHHV-6B	84	1.15	0.36	−2.01
vHHV-6B	68	2.54	1.10	−2.39
iciHHV-6B (USA)	57	0.76	0.29	−2.20
iciHHV-6B (Europe)	21	0.34	0.24	−1.19
vHHV-6B (USA)	35	1.78	0.97	−1.74
vHHV-6B (Asia)	24	0.89	0.71	−0.79
vHHV-6B (Africa)	7	2.29	2.13	−0.41

iciHHV-6A/iciHHV-6B, inherited chromosomally integrated HHV-6A and HHV-6B; vHHV-6A/vHHV-6B, exogenous HHV-6A and HHV-6B viruses.

As for HHV-6B, nucleotide diversity was lower than for HHV-6A, and *D* values were definitely negative (Table 1). Because a large enough number of sequences were available for some locations, analysis was repeated for genomes deriving from different geographic areas. This confirmed that iciHHV-6B from Europe and the USA had low diversity and negative *D* values (Table 1). In accordance with STRUCTURE inference (Fig. 4A), these populations most likely underwent one or more bottlenecks followed by population expansion. Among exogenous viruses the highest diversity was observed in Africa and the lowest in Asia (Table 1). Tajima’s *D* was negative for viruses sampled in all locations, however less so for African sequences. This finding, together with the high diversity levels suggests moderate population expansion in Africa, whereas the results of Asian sequences are most likely influenced by the bottleneck experienced by the viral population with high HHV-6B-3 ancestry component. Data from the USA are more difficult to interpret due to the presence of different viral populations, as assessed by STRUCTURE (Fig. 4A).

## 4. Discussion

Roseolovirus infections are ubiquitous and pose important burdens on human health (Hill and Zerr 2014; Tesini, Epstein, and Caserta 2014). Genetic analyses of the population structure of these viruses became possible only recently, given the increased number of available complete genomes and recognition of the complexities of germline chromosomal integration (Tweedy et al. 2016; Zhang et al. 2017; Greninger et al. 2018a,b;Telford, Navarro, and Santpere 2018). Herein, we used these sequence data to investigate the evolutionary relationships between HHV-6A and HHV-6B, as well as to analyze their genetic diversity and geographic structure (or lack thereof).

In line with previous data, all analyses clearly differentiated HHV-6A and HHV-6B sequences (Tweedy et al. 2016; Zhang et al. 2017; Greninger et al. 2018a,b; Telford, Navarro, and Santpere 2018). STRUCTURE analysis estimated that HHV-6A genomes diverged less from a common ancestral population than HHV-6B genomes. Thus, HHV-6B genomes experienced stronger drift, possibly because of a bottleneck that resulted in loss of diversity, with subsequent population expansion. This view is also supported by calculation of nucleotide diversity and Tajima’s *D*. Based on the plausible assumption that iciHHV-6A/iciHHV-6B have the same mutation rate as the human genome, Zhang et al. (2017) estimated that the integration event of a subset of identical iciHHV-6B in Europeans occurred around 24,000 years ago. It follows that the events that led to the divergence of HHV-6A and HHV-6B viruses occurred in a distant past.

Although the number of analyzed individuals is still limited and biased in terms of origin, estimates indicated that the frequency of iciHHV-6B is higher than that of iciHHV-6A in several populations (Clark 2016; Tweedy et al. 2016). At present, it is unknown which fraction of iciHHV-6A/iciHHV-6B derive from founder effects. Beside the aforementioned European iciHHV-6B sequences, other authors have indicated that multiple integrated sequences (either iciHHV-6A or iciHHV-6B) were inherited from a common human ancestor (Kawamura et al. 2017; Zhang et al. 2017). Data herein do not address this question, nor elucidate the frequency with which HHV-6A/HHV-6B can integrate and be transmitted vertically. STRUCTURE data however indicate that currently sequenced iciHHV-6A derive from viral populations that experienced limited drift compared to iciHHV-6B and are, therefore, most likely older, in agreement with previous estimates (Tweedy et al. 2016). Indeed, iciHHV-6A are diverse and possibly represent a snapshot of integration events that involved extinct or still unsampled viral populations. Extant exogenous HHV-6A viruses, especially those sampled outside Africa, have a large fraction of their ancestry deriving from a population that is not represented in iciHHV-6A. This does not necessarily imply that modern HHV-6A viral populations have reduced integration efficiency, but might instead reflect a sampling bias. Indeed, few HHV-6A sequences (either exogenous or integrated) are available, and it is thus unknown to which degree they are representative of the viral genomes transmitted or circulating in different geographic areas, which are, moreover, unequally sampled (see below). However, the situation observed for HHV-6A is the opposite than that of HHV-6B, whereby integrated copies reflect viral populations responsible for active infections in the same areas. This observation is also consistent with a more recent origin of HHV-6B compared to HHV-6A (Tweedy et al. 2016). Among exogenous HHV-6B viruses, we found that sequences sampled in Africa had the highest nucleotide diversity and a value of Tajima’s *D* relatively close to zero, suggesting constant population size. STRUCTURE analysis indicated that African viral genomes also display the highest proportions of low-drift ancestry components. Overall, these data hint at a possible African origin of circulating exogenous HHV-6B populations, as previously hypothesized for HHV-6A (Tweedy et al. 2016).

The limited sample of exogenous HHV-6A genomes makes it difficult to formulate any hypothesis on their origin. Likewise, only one iciHHV-6B sequence was available from an individual of African ancestry and no iciHHV-6A has been identified in Sub-Saharan Africa to date. This likely reflects the reported lower prevalence of iciHHV-6A and iciHHV-6B in African populations (Clark 2016; Tweedy et al. 2016). Our data do not explain geographic differences in the frequency of integrated viral genomes, which is lower in both African and Asian populations than it is in Europeans and North Americans (Clark 2016; Tweedy et al. 2016). However, for both iciHHV-6A and iciHHV-6B, European and American sequences tend to have high proportions of ancestry from viral populations that experienced considerable drift. A possible interpretation is that environmental, behavioral, demographic, or genetic factors increased infection rates among early Europeans and consequently determined faster changes in viral genetic composition and higher chances for integration. The testing of this and other hypotheses on the geographic origin and spread of HHV-6A/HHV-6B will necessarily require the sequencing of additional integrated and exogenous genomes.

For these very reasons, our study has limitations that include the relatively low number of available HHV-6A sequences and the skewed sampling of both HHV-6A and HHV-6B in terms of geographic locations. Several geographic areas and human ethnic groups or communities are not represented at all or are represented by very few sequences (e.g. Central and Southern Asia, Native Americans, Native Austronesians). The paucity of data from Africa was aforementioned and sampling in Asia is also relatively sparse. Nonetheless, twenty-four HHV-6B exogenous viruses were available for Japan, where the virus represents the second most common cause of infection-related encephalitis (Hoshino et al. 2012). The proportions of ancestry components of most of these viruses were very different from the majority of those sampled in the USA. However, it should be noted that the American samples all derived from a single study that recruited patients in a single location (Rochester, New York State) (Greninger et al. 2018a). Thus, it is unclear to which degree the available sequences are representative of viruses being transmitted in the USA and in Japan.

Another caveat of this study lies in the different methodologies that were applied to sequence both integrated and exogenous viral genomes, which in turn generated sequences with different levels of quality, completeness, and coverage. However, we based all analyses on PIs with sequence information available for more than half of the samples, suggesting that sequencing errors should minimally affect the overall results of the analyses.

We should add that, in contrast to most viral evolutionary analyses, investigation of HHV-6A and HHV-6B has an additional complexity related to the intrinsically different replication process of the integrated and exogenous viral copies. Viral genome replication is in fact performed by distinct enzymes, with different mutation rates, which clearly has a bearing on evolutionary inference. Moreover, several aspects of chromosome integration are still poorly understood, but may affect the composition of extant iciHHV-6A/iciHHV-6B populations. These include the actual frequency of germline integration, the stability of integrated genomes, and the effects on host fitness. Indeed, telomeres carrying integrated viral genomes were reported to be shorter than the other telomeres in somatic cells, possibly resulting in instability of both the host and the integrated viral genomes (Prusty, Krohne, and Rudel 2013; Huang et al. 2014; Zhang et al. 2016). Whether this effect or others, such as viral reactivation or expression of viral genes, modulate host fitness is still unclear (Clark 2016; Collin and Flamand 2017). The association between the presence of iciHHV-6A/iciHHV-6B and cardiac disease has been aforementioned. Additional data, although mostly based on small cohorts, indicate that iciHHV-6A/iciHHV-6B may represent a risk factor in the development of other diseases (Pellett et al. 2012). Notably, these studies did not distinguish iciHHV-6A and iciHHV-6B, raising the possibility the two integrated viruses have distinct effects (if any) on human fitness. Likewise, for exogenous viral genomes, biases in the available samples of HHV-6A and HHV-6B genomes may not only depend on geographic origin but also on other factors. For instance, because most HHV-6B exogenous viruses were sampled from children with acute infections who visited medical infrastructures (Greninger et al. 2018a,b), the possibility exists that viral genomes are commonly sequenced from the most severe cases. Although clinical severity is most likely the results of a number of factors, viral determinants might also play a role and eventually skew the available sample towards more virulent viral populations.

Although HHV-6A and HHV-6B display about 90 per cent interspecies genome sequence identity, in vitro studies showed that HHV-6A, but not HHV-6B, productively infects glial cells (Donati et al. 2005; Harberts et al. 2011; Reynaud et al. 2014). In mouse models, HHV-6A DNA persists for months in the brain, whereas HHV-6B DNA levels decreased rapidly after infection (Reynaud et al. 2014). HHV-6A and HHV-6B also differ in terms of sensitivity to type I interferons, distribution in human tissues, immunological properties, and in other biomedical aspects (Jaworska, Gravel, and Flamand 2010; Ablashi et al. 2014). It is logical to assume that such differences are caused by viral genetic determinants. Our data indicate that, in both viral species, both integrated and exogenous viral genomes have different ancestry components, partially depending on geographic location. It would be extremely important to determine whether such different ancestry composition accounts for the geographic diversity of clinical symptoms and epidemiology. More generally, future efforts to determine and map the genetic determinants of HHV-6A/HHV-6B phenotypes will be pivotal to the understanding of the pathogenic potential of these ubiquitous infectious agents.

## Data availability

A list of NCBI accession number of all sequences analyzed is reported in Supplementary Table S1. The alignment of 181 HHV-6A/HHV-6B genomes is available as Supplementary Text S1.

## Funding

This work was supported by the Italian Ministry of Health (grant number RC 2019 to M.S., RC 2018-2019 to D.F.).

**Conflict of interest:** None declared.

## Supplementary Material

veaa035_Supplementary_Data
