# Supplementary figures and images for: Evolutionary analysis of exogenous and integrated HHV-6A/HHV-6B populations
Source: Virus Evol. 2020 Apr 30;6(1):veaa035. doi: 10.1093/ve/veaa035 (PMC7293831; doi:10.1093/ve/veaa035)

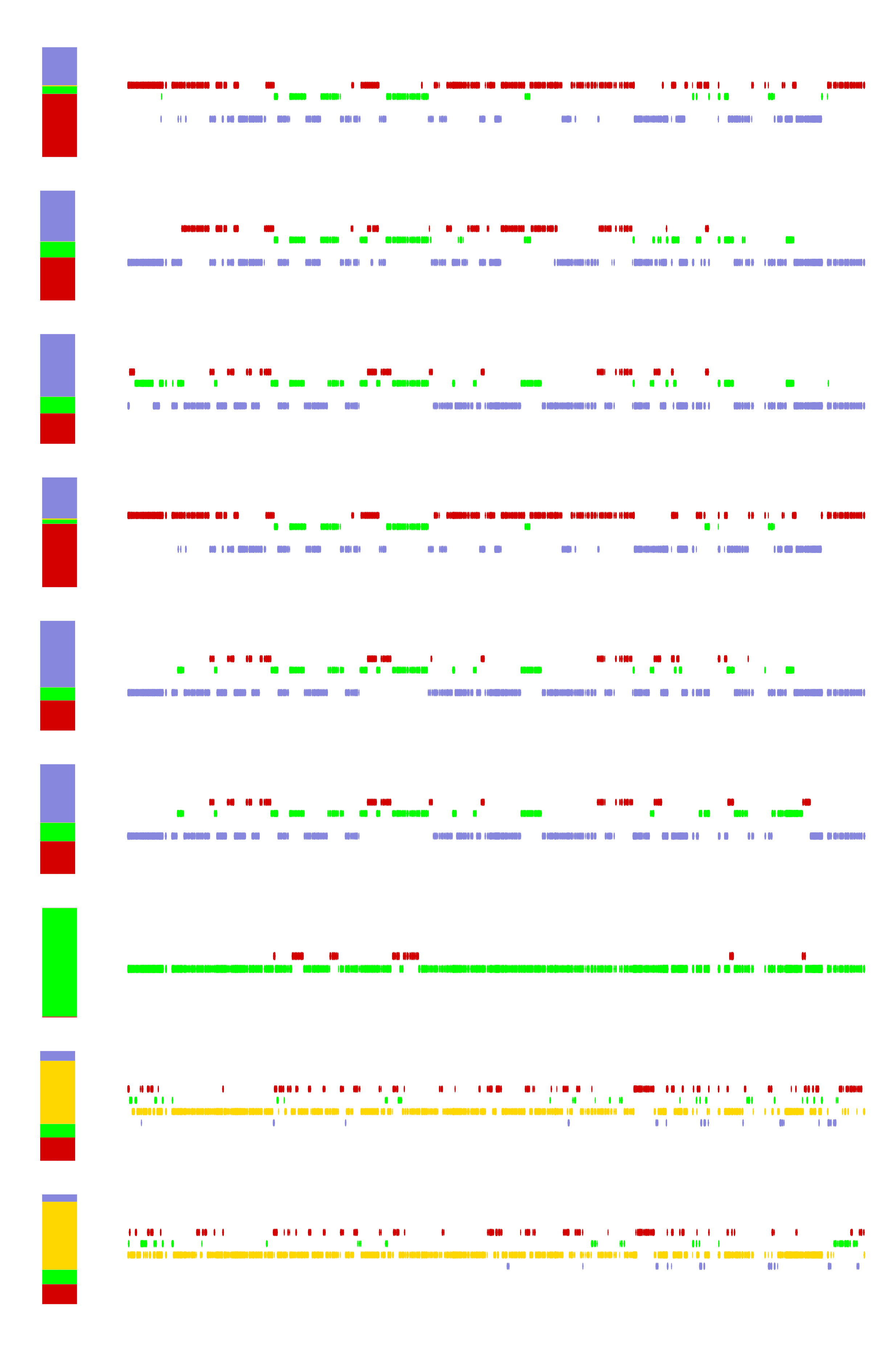

Supplement: veaa035_Supplementary_Data [file veaa035_supplementary_data.png]
